# Optimization of the Froth Flotation Process for the Enrichment of Cu and Co Concentrate from Low-Grade Copper Sulfide Ore

**DOI:** 10.3390/ma18153704

**Published:** 2025-08-06

**Authors:** Michal Marcin, Martin Sisol, Martina Laubertová, Jakub Kurty, Ema Gánovská

**Affiliations:** Institute of Earth Resources, Faculty of Mining, Ecology, Process Control and Geotechnologies, Technical University of Košice, Letná 1/9, Košice-Sever, 042 00 Košice, Slovakia; michal.marcin@tuke.sk (M.M.); martin.sisol@tuke.sk (M.S.); jakub.kurty@tuke.sk (J.K.); ema.ganovska@tuke.sk (E.G.)

**Keywords:** froth flotation, copper concentrate, cobalt concentrate, sulfide, low-grade ore

## Abstract

The increasing demand for critical raw materials such as copper and cobalt highlights the need for efficient beneficiation of low-grade ores. This study investigates a copper–cobalt sulfide ore (0.99% Cu, 0.028% Co) using froth flotation to produce high-grade concentrates. Various types of surfactants are applied in different ways, each serving an essential function such as acting as collectors, frothers, froth stabilizers, depressants, activators, pH modifiers, and more. A series of flotation tests employing different collectors (SIPX, PBX, AERO, DF 507B) and process conditions was conducted to optimize recovery and selectivity. Methyl isobutyl carbinol (MIBC) was consistently used as the foaming agent, and 700 g/L was used as the slurry density at 25 °C. Dosages of 30 and 100 g/t^1^ were used in all tests. Notably, adjusting the pH to ~4 using HCl significantly improved cobalt concentrate separation. The optimized flotation conditions yielded concentrates with over 15% Cu and metal recoveries exceeding 80%. Mineralogical characterization confirmed the selective enrichment of target metals in the concentrate. The results demonstrate the potential of this beneficiation approach to contribute to the European Union’s supply of critical raw materials.

## 1. Introduction

The global demand for critical raw materials (CRMs) has intensified in recent years due to their essential role in the development of green technologies, digital infrastructure, and advanced manufacturing. The European Union (EU) has identified a range of CRMs that are economically important yet subject to supply risk, including cobalt, antimony, and rare earth elements often associated with base metal deposits such as copper ores [1].

Cobalt is a critical raw material widely recognized for its high-performance characteristics and is extensively used across various sectors, including machinery, electronics, electric power, and construction. However, its primary application in recent years has shifted towards the production of lithium-ion (Li-ion) rechargeable batteries, which are fundamental to high-technology devices such as smartphones, tablets, and laptops [2,3]. More importantly, the global demand for cobalt is now predominantly driven by the rapidly expanding electric vehicle (EV) sector. According to industry analyses, the rechargeable battery industry currently accounts for over 50% of global cobalt consumption. This trend is projected to intensify due to increasing governmental policy support, climate change mitigation goals, and strategic efforts to transition towards low-carbon technologies. As a result, global cobalt consumption is anticipated to triple within the next decade. According to data from the United States Geological Survey, the majority of economically recoverable cobalt reserves are located in sediment-hosted copper–cobalt (Cu–Co) deposits in the Democratic Republic of Congo (DRC), where cobalt is primarily obtained as a by-product of copper sulfide ore processing [4,5,6,7].

Copper is a vital industrial metal known for its excellent ductility, electrical and thermal conductivity, and recyclability. It is extensively used across various sectors, including electrical engineering, renewable energy systems, and machinery manufacturing. Globally, copper ranks third in production and consumption after steel and aluminum, underscoring its strategic importance in modern technological and industrial development [8,9].

The mining industry produces vast amounts of tailings, typically stored in large tailings storage facilities, which pose challenges related to dam stability and environmental risks, especially for sulfide-rich materials. Despite these risks, tailings can contain valuable metals, making them potential sources of secondary raw materials [10,11,12].

Flotation is a widely employed and cost-effective technique for enhancing the grade of sulfide ores, significantly contributing to the reduction of downstream metallurgical processing costs. Numerous studies have focused on copper sulfide flotation, addressing aspects such as process mineralogy, flotation methodologies, and reagent selection [13,14].

The flotation process is mostly regulated by the surface chemistry of separating minerals. The adsorption of collectors on sulfide minerals occurs through two main mechanisms: chemical adsorption, where chemisorbed metal xanthate forms on the mineral surface, and electrochemical adsorption, where oxidation products create hydrophobic species that adhere to the surface. These processes enhance the mineral’s floatability in flotation systems [15]. Sulfide minerals float only under appropriate redox conditions, with Eh and pH values of the pulp controlling mineral–collector reactions and thus influencing flotation performance [16].

Research on copper sulfide flotation reagents has primarily concentrated on collectors. Beyond traditional xanthates, novel collectors such as heterocyclic compounds and phosphates have been explored [17]. Recent studies have also increasingly investigated sulfide flotation inhibitors, including sodium phytate and xanthan gum [18]. Additionally, the influence of unconventional agents such as anionic polyacrylamide, n-dodecane, and residual xanthate on sulfide flotation has been reported [19,20,21]. Studies confirm that the synergistic effect of novel collectors, depressants, and frothers enables better control of the flotation process and contributes to increased recovery and improved concentrate quality in the treatment of complex sulfide ores [22,23,24]. 

This study focuses on the beneficiation potential of a low-grade, low-sulphide copper ore, with the overarching objective of developing an effective processing strategy that maximizes the recovery of copper and associated critical raw materials—particularly cobalt—through froth flotation. The main objective is to produce economically valuable, high-grade concentrates suitable for further metallurgical processing, while demonstrating that even low-sulphide ore bodies can serve as viable sources of strategic metals. To achieve this, the study systematically investigates the influence of various flotation reagents and operational parameters on metal recovery and concentrate quality. This integrated approach provides a scientific basis for the efficient processing of underexplored ore types and supports future efforts in diversifying the EU’s supply of critical raw materials.

The novelty of this study lies in its comprehensive investigation of a largely unexplored, low-sulfide copper deposit located in Central Europe—an ore type that has received minimal attention in scientific literature. Unlike conventional studies that primarily focus on high-sulfide or polymetallic deposits, this research targets a low-grade (0.99% Cu), low-sulfide ore and explores its potential for economic beneficiation using froth flotation. Through the application of tailored reagent regimes, selective flotation strategies, and advanced analytical techniques, the study demonstrates that even such geologically less favorable ores can produce high-grade concentrates with significant recovery rates. This provides a novel and practical pathway for enhancing the domestic supply of critical raw materials (CRMs) in the European Union, reducing dependency on imports and contributing to the strategic goals of raw material security and sustainability. Additionally, the integration of SEM-EDX mineralogical characterization provides a deeper understanding of CRM associations within the ore matrix, which is essential for the future development of efficient recovery strategies.

## 2. Materials and Methods

### 2.1. Ore Sample 

The raw material utilized in this study was composed of primary ore magnesite, with subordinate quantities of talc. Importantly, this ore body also contains trace amounts of economically significant metals, namely cobalt (Co), copper (Cu), and gold (Au).

These metals occur as accessory mineral phases within a magnesite–talc lens. The occurrence of cobalt, copper, and gold is classified as secondary or accessory, and they are not currently exploited as primary resources. Despite their non-recovery, recent mineralogical and geochemical analyses suggest that their aggregate gross value constitutes approximately 40% of the total economic value of the mined raw material. This indicates a potentially valuable opportunity for secondary metal recovery during magnesite processing, particularly in the context of increasing global demand for critical raw materials such as cobalt and copper.

The strategic evaluation of these by-product metals could lead to enhanced resource utilization, increased economic efficiency, and alignment with sustainable mining practices.

The intimate intergrowth of valuable minerals with gangue necessitates efficient liberation prior to separation. The raw feed material, initially sized between 0 and 320 mm, underwent a two-step comminution process. It was first reduced using a jaw crusher (BB500, Retsch GmbH, Haan, Germany), then finely ground in a laboratory ball mill (TM 500, Retsch GmbH, Haan, Germany) to ensure adequate release of target minerals for processing.

A bulk ore sample was crushed and milled for experimental testing. To ensure representativeness, the sample was homogenized and quartered using the coning and quartering technique. Representative subsamples were then obtained by riffle splitting for subsequent flotation and analytical procedures. All samples were stored in airtight polyethylene containers under dry, ambient conditions to prevent oxidation or contamination prior to testing.

### 2.2. Sample Characterization

The particle size distribution “Mastersizer analysis” of the ore sample was analyzed using a Malvern Mastersizer 3000 laser diffraction instrument (Malvern Instruments Ltd., Malvern, UK). A representative subsample was prepared by dispersing the material in demineralized water to form a slurry with a solids concentration of approximately 30 wt.%, maintained at ambient temperature. To ensure thorough dispersion of the mineral particles, the suspension was stirred at 690 rpm for 10 min. Each subsample was analyzed three times, and the final particle size distribution was calculated as the average of 15 individual measurements.

Samples were pulverized to achieve 85% passing below 0.075 mm. Mineral concentrates were analyzed using certified analytical protocols tailored to each concentrate type. Quantitative elemental analysis was conducted via X-ray fluorescence (XRF) (SPECTRO Analytical Instruments GmbH, Kleve, Germany) and atomic absorption spectroscopy (AAS) (iCE 3300 AA, Thermo Fisher Scientific, Grand Island, NY, USA), depending on elemental requirements and detection limits.

Selected feed and concentrate samples were characterized using scanning electron microscopy (SEM) (MIRA 3 FE-SEM, TESCAN, Brno, Czech Republic), equipped with a Schottky field emitter, three-lens Wide Field Optics™, and an energy-dispersive X-ray detector (EDX) (Oxford Instruments, Abingdon, UK).

X-ray powder diffraction (XRPD) was employed to characterize the crystalline phases present in the samples. Diffraction patterns were recorded at ambient temperature using a Bruker D8 Advance diffractometer equipped with Cu Kα radiation and configured in the Bragg–Brentano geometry. The instrument operated at 40 kV and 40 mA, scanning over a 2θ range of 5° to 100°. Phase identification was performed using reference patterns from the Inorganic Crystal Structure Database (ICSD).

The chemical analysis of the raw ore is shown in Table 1. The chemical analysis indicates that the ore is a valuable secondary raw material, mainly due to the content of Cu (0.99%), Co (0.028%), and other elements such as Mg (25.5%) and Fe (3.79%).

### 2.3. Froth Flotation Tests

Flotation tests were carried out using an automated laboratory froth flotation unit equipped with a 2 L and 4 L Denver-type cell (Outotec GTK LabCell, Metso, Espoo, Finland). All experiments were conducted at a controlled temperature of 25 °C. The slurry was introduced into the flotation cell along with a pre-weighed sample. Agitation was initiated at 1100 rpm, followed by the sequential addition of flotation reagents.

The slurry was conditioned for 5–25 min, with the duration tailored to the type and number of reagents used. After conditioning, air was introduced at a constant rate of 5 L·min^−1^, regulated electronically to maintain uniformity across all tests. After flotation, the concentrates were collected, dried, and assayed for Zn, Pb, and Cu using atomic absorption spectroscopy (AAS), and metal recoveries in the concentrates were calculated using Equation (1).

(1) $$Metal\;recovery\;\varepsilon =\frac{Cc}{Ff}\times 100\%$$

where *C* and *F* represent the dried weights of the flotation concentrate and feed, respectively, whereas *c* and *f* are the metal grades in the concentrate and feed, respectively. All reagents used for Froth flotation tests are summarized in Table 2.

**Figure 1:**
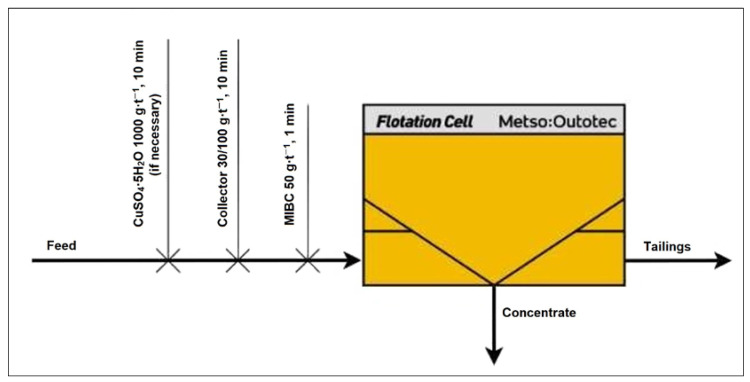
Flowchart of the flotation procedure, made by HSC Chemistry 10 software (10.5.4 vision), Modul HSC Sim [25].

Figure 1 illustrates the flotation procedure applied during the preliminary experiments. 

Both collective and selective flotation experiments were carried out to evaluate the beneficiation potential of the investigated low-grade copper–cobalt ore. In the collective flotation stage, copper and cobalt minerals were floated together using a single-stage reagent scheme, aiming to maximize overall metal recovery. These procedures allowed for a comparative analysis of the efficiency of different reagent schemes and flotation strategies in producing either bulk or metal-specific concentrates.

Based on the results of the experiments conducted, a two-stage selective flotation procedure was developed to separately recover copper and cobalt into distinct concentrates. In the first stage, copper was selectively flotated using the PBX (100 g·t^−1^) collector in the presence of copper(II) sulfate (1000 g·t^−1^) as an activator. After completion of copper flotation, the process was continued with a 10-min conditioning period.

**Figure 2:**
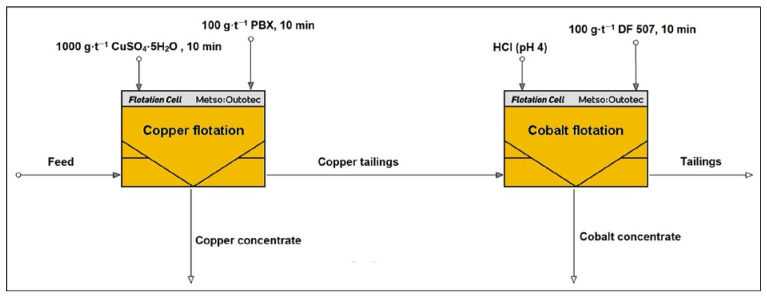
Schematic diagram of selective flotation of copper and cobalt concentrates, made by HSC Chemistry 10 software (10.5.4 vision), Modul HSC Sim [25].

Subsequently, the slurry pH was reduced to approximately 4.0 using hydrochloric acid (HCl) to enhance cobalt activation and collector interaction. The DF 507B (100 g·t^−1^) collector was then added to initiate cobalt flotation. This two-step approach allowed for the sequential recovery of copper and cobalt into separate concentrates, thereby improving selectivity and reducing cross-contamination between the metals. A schematic diagram illustrating the selective flotation flowsheet is presented in Figure 2.

## 3. Results and Discussion

### 3.1. Grain Size Analysis

**Figure 3:**
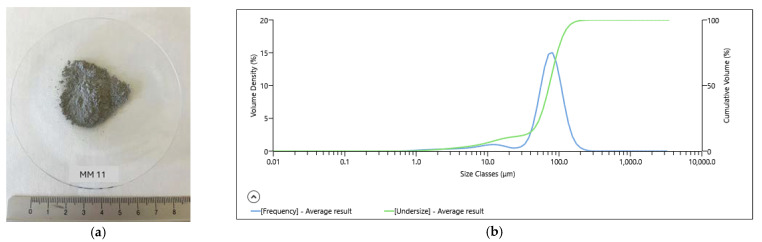
The ore sample (**a**); particle size distribution of the ore (**b**).

A view of the different sizes of the ore sample particles (without magnification) was observed (Figure 3a).

The particle size distribution (PSD) of the ore sample, obtained by “Mastersizer analysis”, is represented in Figure 3b. According to the laser scattering data, the particle size distribution of the sample ranged from 0.1 to 1000 μm, with a P_80_ of 102 μm, with P_50_ less than 72 μm.

### 3.2. SEM-EDX Analysis 

The selected samples and feed material were analyzed using a scanning electron microscope (SEM), and an energy-dispersive X-ray (EDX) detector.

**Figure 4:**
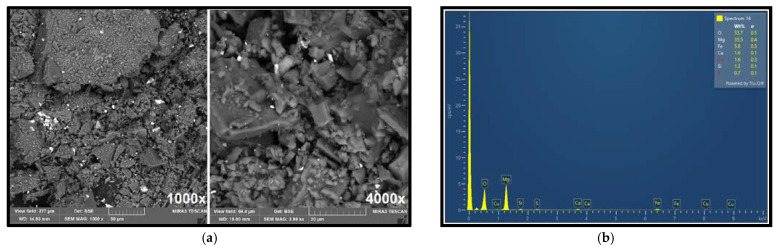
Results of SEM analysis of the ore sample (**a**) 1000× magnification and 4000× magnification; (**b**) EDX analysis of the ore.

Figure 4a shows the morphology of the ore particles observed via SEM imaging at 1000× and 4000× magnification, showing different particle shapes and sizes. Figure 4b presents the elemental composition obtained by EDX analysis. The surface elemental composition (35.5% Mg, 5.8% Fe, 1.6% Ca, and 1.6% Cu) determined by EDX closely corresponds with the ore composition previously established by atomic absorption spectroscopy (AAS). EDX analysis further confirmed the presence of copper in the raw ore, with a measured concentration of approximately 1.6%, supporting the estimated average content of around 1%.

### 3.3. XRD Analysis

**Figure 5:**
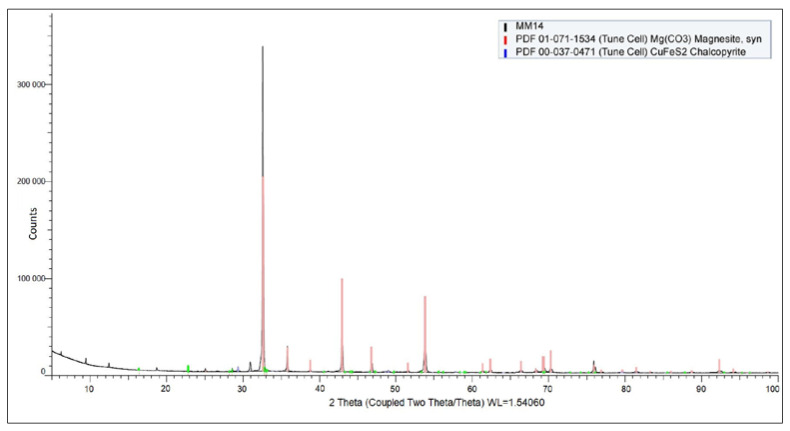
XRD spectrum of the sulfide ore sample.

Figure 5 shows the XRD diffraction pattern of the input sample. Magnesite was confirmed as the predominant mineral phase in the sample.

Additionally, the presence of chalcopyrite is highly probable, although detected only in trace amounts. The diffraction pattern also indicated minor signals corresponding to an unidentified phase, which could potentially originate from residual organic matter or complex high-molecular-weight compounds. 

### 3.4. Forth Flotation Tests

Prior to each experiment, a specific flotation procedure was selected based on the test objectives. The flotation tests were performed in cells with volumes of 2 L and 4 L, with continuous agitation of the slurry at a fixed speed of 1100 rpm. The slurry density was maintained at 700 g/L across all experiments, and the temperature was kept constant at approximately 25 °C. The pH varied depending on the conditions of each individual test.

A total of 27 flotation experiments were planned, each with an approximate duration of 40 min. Methyl isobutyl carbinol (MIBC) was consistently used as the frother in all tests. Other reagents such as collectors, activators, depressants, and dispersants were selected based on the specific flotation protocol applied in each test.

Upon completion of each flotation test, both the froth (concentrate) and tailings (waste) were collected, filtered, and dried in a laboratory drying machine before being submitted for further analysis.

#### 3.4.1. Effect of Collector Type on the Recovery of Metals

**Figure 6:**
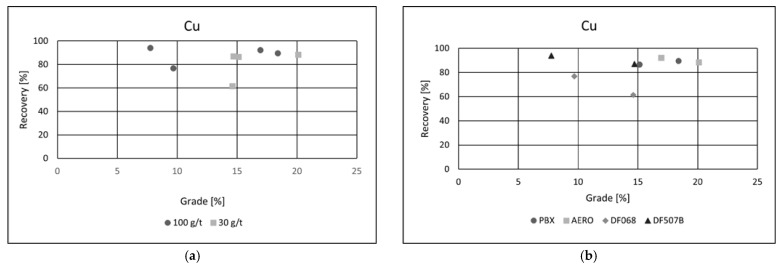
Grade/recovery diagrams of copper recovery with respect to collector dosage (**a**) and collector type (**b**).

In all flotation experiments conducted on the ore, the recovery of each target metal was calculated separately. After flotation, the mass of the froth (concentrate) product was measured to determine the yield. Based on the analytical data, grade–recovery curves were generated to assess metallurgical performance across different flotation conditions. Effects of collector dosage and type of collector were examined. The results (Figure 6a,b) indicate that both the recovery and grade of copper in the concentrate were achieved at technically satisfactory levels, demonstrating the effectiveness of the applied flotation conditions [26,27].

This level of enrichment is considered industrially viable for further metallurgical processing. A clear trend was observed in response to increasing collector dosage: higher collector concentrations led to an improvement in copper recovery, attributed to enhanced hydrophobicity and increased attachment of copper-bearing particles to air bubbles.

However, this increase in recovery was accompanied by a slight reduction in copper grade. This inverse relationship is commonly observed in flotation systems and is likely due to the non-selective adsorption of the collector at higher dosages, which promotes the entrainment or flotation of gangue minerals alongside copper particles. As a result, while metal recovery benefits from increased reagent input, the concentrate purity may be compromised. This balance between recovery and grade highlights the importance of optimizing reagent dosages to achieve the desired compromise between metallurgical efficiency and concentrate quality in copper flotation. 

**Figure 7:**
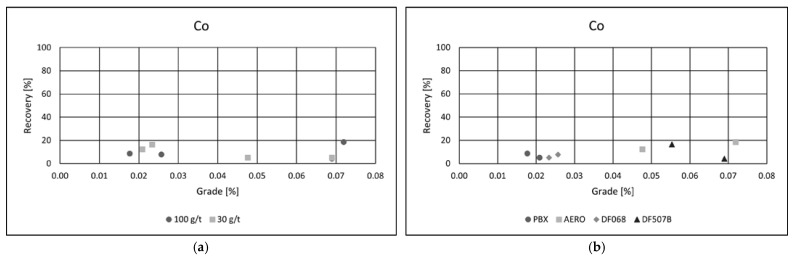
Grade/Recovery diagrams of cobalt recovery with respect to collector dosage (**a**) and collector type (**b**).

Collector type had a notable impact on flotation performance. Among the tested reagents, PBX and AERO produced the best results, achieving both high copper recoveries and superior concentrate grades. Their effectiveness is likely due to stronger and more selective adsorption onto copper-bearing minerals, enhancing separation efficiency. In comparison, the other two collectors yielded similar recoveries but resulted in significantly lower copper grades, suggesting reduced selectivity and increased entrainment of gangue minerals. These results emphasize the importance of choosing appropriate collectors to optimize both recovery and concentrate quality in copper flotation. Grade/recovery diagrams of cobalt flotation performance are shown in Figure 7a,b.

Although cobalt recoveries did not reach the same levels as those observed for copper, a substantial enrichment of cobalt in the concentrate was achieved. In the most successful flotation test, the cobalt content in the concentrate increased by more than twenty times compared to its concentration in the raw ore, indicating effective upgrading potential.

As with copper, a collector dosage of 100 g·t^−1^ was found to be optimal for cobalt flotation, providing a favorable balance between recovery and selectivity. Among the collectors tested, AERO and DF 507B delivered the best performance, achieving the highest cobalt grades in the final concentrate. These collectors likely demonstrated a strong adsorption affinity for cobalt-bearing minerals, contributing to enhanced flotation selectivity.

The best result was obtained using the AERO collector at a dosage of 100 g·t^−1^, yielding a cobalt grade of 0.07% and a recovery of 18.5%. While the overall recovery remained moderate, the high enrichment factor highlights the potential of flotation to concentrate cobalt efficiently, making it a viable method for recovering cobalt as a by-product from complex polymetallic ores.

#### 3.4.2. Effect of Activator on the Recovery of Metals

**Figure 8:**
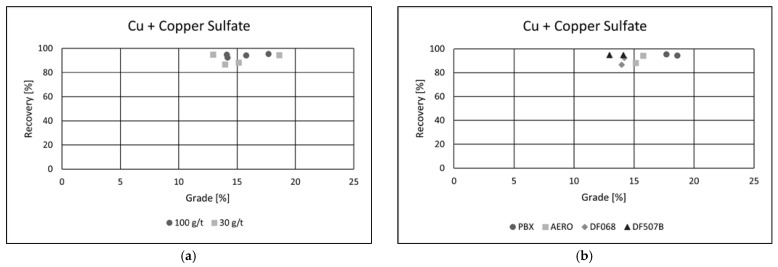
Grade/recovery diagrams of copper recovery with respect to collector dosage (**a**) and collector type (**b**).

In the subsequent tests, a 4-L flotation cell was used, while the slurry density and other flotation parameters remained unchanged. To improve the quality of the resulting concentrates, copper(II) sulfate (CuSO_4_·5H_2_O) was added as an activator at a dosage of 1000 g·t^−1^. The copper sulfate was introduced into the slurry during conditioning, 10 min prior to the addition of collectors, to enhance the surface reactivity of target minerals and promote collector attachment. The addition of copper(II) sulfate (CuSO_4_·5H_2_O) as an activator led to a noticeable improvement in flotation performance across all tested collectors (Figure 8a,b).

The differences in collector efficiency became less pronounced, and a general enhancement in copper recovery was observed. In the best-performing test, copper recovery exceeded 95%, indicating effective activation of the copper-bearing minerals and improved collector interaction.

The collector dosage showed minimal variation in its influence under these conditions, suggesting that the activation effect dominated the flotation response. Among all the reagents, the PBX collector exhibited the highest efficiency, delivering the best overall performance.

**Figure 9:**
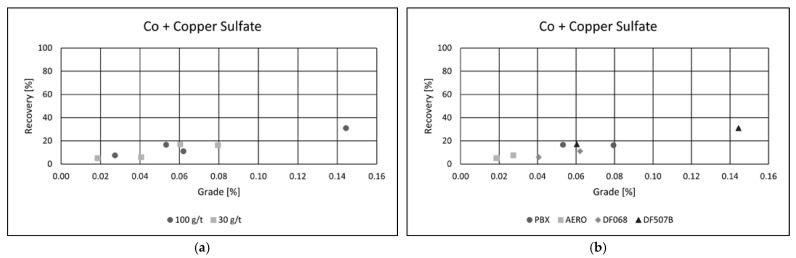
Grade/recovery diagrams of cobalt recovery with respect to collector dosage (**a**) and collector type (**b**).

In the test using PBX at a dosage of 100 g·t^−1^, the flotation process achieved a copper recovery of 95.47%, with a final concentrate grade of 17.69% Cu. These results highlight the effectiveness of copper sulfate as an activator and confirm PBX as the most suitable collector under the tested conditions for maximizing both recovery and concentrate quality. The addition of copper(II) sulfate as an activator had a clear positive effect on cobalt flotation performance (Figure 9a,b).

Both the recovery and grade of cobalt in the final concentrate improved significantly following activation. Although cobalt recoveries remained lower than those achieved for copper, the enhancement in flotation efficiency was clearly observed.

Among the tested collectors, PBX and DF 507B showed the most favorable results, each achieving cobalt recoveries of approximately 20% and concentrate grades exceeding 0.05% Co. The highest performance was recorded using the DF 507B collector at a dosage of 100 g·t^−1^, where cobalt recovery reached 30.92%, and the cobalt content in the concentrate increased to 0.14%.

These findings suggest that copper sulfate activation enhances the surface reactivity of cobalt-bearing minerals, facilitating better adsorption of collectors and more efficient separation from gangue. The results also confirm the suitability of DF 507B and PBX as selective collectors for cobalt under the tested flotation conditions.

#### 3.4.3. Effect of Selective Flotation on the Recovery of Metals

The designed two-stage selective flotation experiment successfully produced a primary froth product, intended to be a copper concentrate. This concentrate achieved a copper grade of 21.65% with a recovery of 93.77%. Such high copper content reflects excellent performance, corresponding to an enrichment factor of 19.5 compared to the original ore. This indicates the strong efficiency of the flotation process in upgrading copper-bearing minerals.

The primary objective of this experiment, however, was not only to produce a high-quality copper concentrate but also to enhance the recovery and grade of cobalt in the subsequent flotation stage. The second, cobalt-selective concentrate reached a cobalt grade of 1.33%, representing a 28-fold enrichment relative to the feed material. Moreover, cobalt concentrate recovery was recorded at 43%, the highest value achieved throughout the entire experimental campaign.

**Figure 10:**
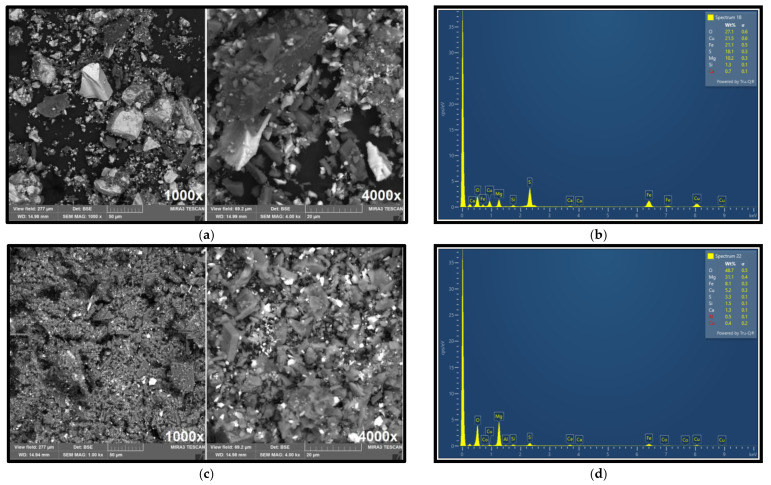
Results of SEM analysis of concentrate sample fraction Cu: 1000× magnification and 4000× magnification (**a**); EDX analysis of the concentrate sample fraction Cu (**b**); SEM analysis of concentrate sample fraction Co: 1000× mag and 4000× mag (**c**); EDX analysis of the concentrate sample fraction Co (**d**).

These results demonstrate the effectiveness of sequential flotation with pH modification and targeted reagent selection in producing separate, high-quality concentrates of copper and cobalt. The methodology offers a promising route for the valorization of cobalt as a by-product in polymetallic ores. Figure 10a,b present the results of SEM and EDX analyses of the selective concentrate obtained from flotation focused on copper recovery.

These analyses clearly demonstrate significant enrichment of copper in the concentrate, with EDX confirming a copper content of 21.65%. This substantiates the efficiency of the flotation process in selectively concentrating copper-bearing minerals.

**Figure 11:**
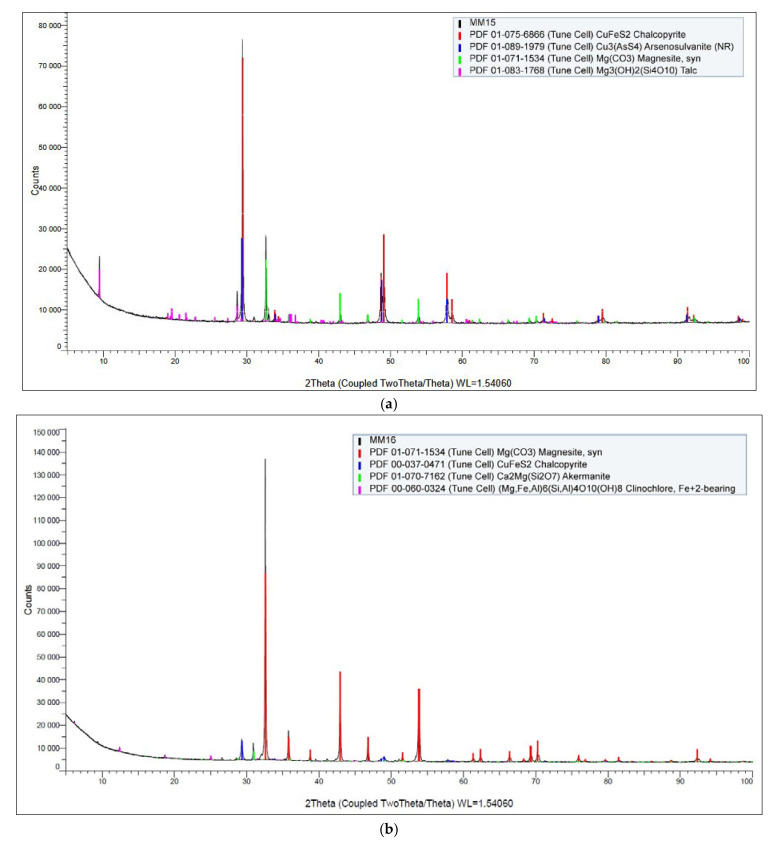
XRD spectrum of the copper concentrate (**a**), and the cobalt concentrate (**b**).

In contrast, Figure 10c,d shows the corresponding SEM and DX analyses for the cobalt-selective concentrate. In this case, cobalt was detected at a concentration of approximately 0.4%, indicating successful enrichment of cobalt relative to its original content in the feed material. These findings support the effectiveness of the sequential flotation strategy in isolating target metals into discrete, high-grade concentrates. Figure 11a,b displays the X-ray diffraction (XRD) patterns of the selective copper and cobalt concentrates, respectively.

In the case of the copper-selective concentrate (Figure 11a), the diffraction analysis clearly confirmed the presence of chalcopyrite (CuFeS_2_) as a dominant crystalline phase, consistent with the elevated copper content observed in previous EDX analysis. This supports the conclusion that copper was effectively concentrated in the form of chalcopyrite through the flotation process.

In contrast, the XRD pattern of the cobalt-selective concentrate (Figure 11b) revealed magnesite (MgCO_3_) as the primary crystalline phase. Despite the successful cobalt concentrate enrichment confirmed by chemical and EDX analysis, cobalt-bearing phases were not explicitly identified in the XRD spectrum. This is likely due to the detection limit of the XRD method, which typically requires a minimum phase concentration of approximately 1% for reliable identification. As a result, cobalt may be present in either amorphous form, finely disseminated phases, or solid solution within other minerals, falling below the resolution threshold of the technique.

The study confirms that flotation recovery of Cu–Co can be significantly improved by optimizing the process based on the flotation characteristics of the ore and flotation products. In addition to this study, there are numerous reports on the flotation of Cu-Co ores. Dehaine et al. [28] used a collector dosage of 30 g/t DTP (sodium O,O-diisobutyl dithiophosphate) which was found to be optimal, achieving copper and cobalt recoveries of 94.2% and 84.1%, with concentrate grades of 25.8% Cu and 2.4% Co. Authors Shi et al. [29] optimized the flotation process based on ore and product mineralogy using a sequence of one rougher, three cleaners, and two scavenger stages that significantly improved Cu and Co grades. Hu [4] described the optimal collector blend of 35% aerofloat and 65% butyl xanthate, which achieved high Cu (91.91%) and Co (86.1%) recoveries while rejecting 87.23% of the ore as tailings. Although isoamyl xanthate improved grades, it reduced recoveries. A collector dosage of 50 g·t^−1^ was selected as cost-effective, maintaining recoveries above 88% for both metals with minimal performance loss. 

## 4. Conclusions

This study demonstrated the feasibility of recovering copper- and cobalt-bearing concentrates from a low-grade sulfide ore using optimized flotation techniques. Through systematic experiments involving reagent selection, dosage optimization, pH control, and activator use, high-quality selective concentrates of both metals were successfully produced. The findings highlight the importance of process design and reagent chemistry in the beneficiation of complex polymetallic ores.

Copper concentrate flotation:

- PBX and AERO collectors were the most effective.
- Optimal dosage: 100 g·t^−1^, with PBX yielding 95.47% recovery and 17.69% Cu grade.
- Addition of copper sulfate (CuSO_4_·5H_2_O) enhanced recovery and stabilized collector performance.

Cobalt concentrate flotation:

- Without activator, cobalt recoveries were low; addition of copper sulfate significantly improved results.
- DF 507B and PBX collectors achieved ~20–30% cobalt recovery and up to 0.14% Co grade.
- Best cobalt result: 30.92% recovery and 0.14% Co grade with DF 507B at 100 g·t^−1^.

Selective flotation approach:

- Developed a two-stage process: copper flotation followed by cobalt flotation after pH adjustment.
- Stage 1 (Cu): 21.65% Cu grade, 93.77% recovery (19.5× enrichment).
- Stage 2 (Co): 1.33% Co grade, 43.0% recovery (28× enrichment).
- pH reduction to ~4 using HCl was critical for cobalt selectivity.

The results confirm that selective flotation with pH control and reagent tailoring is an effective method for the recovery and enrichment of copper and cobalt concentrate from complex ores. This study demonstrates the potential of optimizing flotation techniques to recover critical raw materials from low-grade, low-sulfide Cu–Co ores in the European Union, contributing to domestic CRM supply. The application of both collective and selective flotation enables efficient metal separation. However, limitations include complex mineralogy, low metal content, and the need for pilot-scale validation to confirm industrial applicability.

## Figures and Tables

**Table 1:** Chemical analysis of major elements in the sample ore by the AAS method.

Content (wt.%)	Cu	Co	Mg	Fe	Si	Ca	Residue
**Average**	0.99	0.028	25.5	3.79	1.58	0.93	67.18
**Standard Deviation**	5.3 × 10^−3^	7.5 × 10^−4^	0.59	5 × 10^−3^	3.15 × 10^−2^	1.14 × 10^−2^	-
**Variance**	2.8 × 10^−5^	5.6 × 10^−7^	0.35	2.5 × 10^−5^	9.92 × 10^−4^	1.29 × 10^−4^	-

**Table 2:** Reagents used for froth flotation tests.

Reagent	Dosage	Purity	Purpose	Producer	City/State
	(g·t^−1^)				
SIPX	30; 100	Analytical	Collector	Thermo Fisher Scientific	Ward Hill, MA, USA
PBX	30; 100	Analytical	Collector	Tokyo Chemical Industry Co., Ltd.	Tokyo, Japan
Aero	30; 100	Analytical	Collector	Cytec Industries Inc.	Princeton, NJ, USA
DF 507B	30; 100	Analytical	Collector	FMC Corporation	Harboøre, Denmark
Copper(II) Sulfate (CuSO_4_·5H_2_O)	1000	Analytical	Activator	Sigma-Aldrich	Burlington, MA, USA

## Data Availability

The original contributions presented in the study are included in the article, further inquiries can be directed to the corresponding author.
